# Sex/gender differences in metabolic syndrome among cancer survivors in the US: an NHANES analysis

**DOI:** 10.1007/s11764-023-01404-2

**Published:** 2023-06-22

**Authors:** Adaora Ezeani, Justin B. E. Tcheugui, Tanya Agurs-Collins

**Affiliations:** 1 National Cancer Institute, Rockville, MD 20850 USA; 2 Johns Hopkins School of Medicine, Baltimore, MD 02115 USA

**Keywords:** Metabolic syndrome, Cancer survivorship, Cancer epidemiology, Sex differences, Gender differences

## Abstract

**Background:**

The purpose of this study was to assess the association of metabolic syndrome (MetS) and its individual components in cancer survivors (CS) by gender, in comparison to participants without a history of cancer who have at least one chronic disease (CD) and those without a chronic disease diagnosis (NCD).

**Methods:**

Data from participants 40 years and older (n = 12,734) were collected from the 2011 to 2018 National Health and Nutrition Examination Survey dataset. MetS was defined based on the National Cholesterol Education Program’s Adult Treatment Panel III. Chi-square test and multivariate-adjusted logistic regression was used to assess group comparisons and associations respectively.

**Results:**

Compared to NCD, CS and CD men had increased odds of meeting MetS, OR 2.60 (CI 1.75–3.87) and OR 2.18 (CI 1.59–2.98) respectively. For women, CS and CD participants also had higher odds of meeting MetS criteria compared to their healthy counterparts, OR 2.05 (CI 1.44–2.93) and OR 2.14 (CI 1.63–2.81) respectively. In subgroup analysis by cancer site, CS men with a history of hematologic malignancies (OR 4.88, CI 1.30–18.37) and CS women with cervical cancer (OR 4.25, CI 1.70–10.59) had highest odds of developing MetS, compared to NCD. CS men also showed a strong association with elevated waist circumference, low high density lipoprotein-c, and elevated triglycerides, even by cancer site, but there were no consistent findings among women.

**Conclusion:**

This study indicates that CS men have a strong association with MetS, especially among those with blood-related cancers.

The metabolic syndrome (MetS), a collection of metabolic abnormalities and a proxy of insulin resistance, is linked to type 2 diabetes, cardiovascular disease and cancer [1, 2]. MetS has become a growing public health concern in the United States. Indeed, with the increase in sedentary lifestyle and obesity in the general population [3, 4], the prevalence of MetS is rising, with 25–40% of US population meeting the criteria for diagnosis, depending on the definition and cut-off values used for each factor [5–7]. Epidemiological studies have noted that incidence of MetS differs by sex or gender, for example, being reportedly more prevalent in men than women, but with women experiencing a steeper increase in prevalence [7]. Many components of MetS have been linked to sex- or gender-related factors including hormonal status, socio-economic status, and adoption of unhealthy behaviors [8]. Particularly, there is strong evidence that insulin-resistance and increased abdominal fat have differential effects in men and women [8].

MetS has been shown to influence cancer development and progression, and thus cancer-related mortality [2, 9]. Recent meta-analyses have showed that MetS is associated with several cancers, including colorectal, and bladder cancers in men, and endometrial, post-menopausal breast, and colorectal cancers in women [2, 10]. Given the growing cancer survival rates, due to improved early detection and treatment [11], MetS can also represent a common long-term complication after treatment that affects quality of life. Cancer survivors with MetS are faced with adverse effects, such as atherosclerotic disease or cancer recurrence, which demands proactive assessment and management by healthcare professionals. The purpose of this study is to assess the association between cancer survivors and MetS and its individual components to help determine the risk for cardiovascular disease, secondary cancers, and other sequalae due to MetS. In addition, to compare differences between cancer survivors and non-cancer survivors, this study aims to analyze the association between MetS and its components to participants without a cancer diagnosis, categorized into a healthy group and group with comorbidities.

## Methods

### Study population

The data used in this study were acquired from the US National Health and Nutrition Examination Survey (NHANES), conducted from 2011 to 2018 by the Centers for Disease Control and Prevention [12]. NHANES is a nationwide, population-based cross-sectional study, representing non-institutionalized, civilian US population. It is designed to assess the health and nutritional status of adults and children in the United States. A multistage sampling procedure was used. The current analysis was restricted to adults aged 40 years or older. We excluded participants with a history of childhood cancer (n = 13), defined as cancer diagnosis at 16 years old or younger, because of the growing evidence supporting increased risk of metabolic syndrome in pediatric cancer survivors [13–15]. Pregnant women (n = 14) were excluded due to pregnancy-related metabolic changes and increased waist circumference. Our final analytical sample included 12,734 individuals. The study sample was categorized into three groups or statuses: (1) participants with no history of self-reported medical conditions (NCD), (2) participants with a self-reported history of at least one chronic disease and no history of cancer (CD), and (3) participants with a history of cancer (CS). Participants assigned to the CD group reported a history of medical conditions used to calculate the Charlson Comorbidity Index, a tool used to quantify the burden of comorbidity and evaluate risk of mortality [16, 17]. NHANES interviewers asked participants if they had a professional diagnosis of the following: arthritis, gout, congestive heart failure, heart attack, stroke, a liver condition including hepatitis B and C, kidney disease such as kidney stones and kidney failure, diabetes, and emphysema and/or chronic bronchitis or chronic obstructive pulmonary disease. Participants were assigned to the CD group if they replied “yes” to the question “Has a doctor or other health professional ever told you that you have [condition]?”.

### Metabolic syndrome definition

In accordance with the National Cholesterol Education Program (NCEP) Adult Treatment Panel III (ATP III) guidelines [18], we defined MetS as the presence of at least three of the following: (1) elevated waist circumference (≥ 102 cm for men, ≥ 88 cm for women), (2) low high density lipoprotein (< 40 mg/dL for men and < 50 mg/dL for women) or current treatment for reduced HDL-C, (3) elevated triglycerides (≥ 150 mg/dL) or current treatment for elevated triglycerides, (4) elevated fasting plasma glucose (≥ 100 mg/dL) or current use of diabetes medication, and (5) elevated blood pressure (systolic blood pressure ≥ 130 or diastolic blood pressure ≥ 85) or current use of blood pressure medication.

### Covariates

#### Sociodemographics

Questionnaires were used to obtain information on demographics (age, race, gender, and/or ethnicity), education, and annual family income. Age was categorized into three groups: 40 to 59 years, 60 to 79, and 80 years or older. Gender was categorized as man or woman. It is important to note that NHANES dataset adopts a cisnormative approach where gender and sex are not differentiated, and therefore, both measures are considered concordant in this study. Race/ethnicity was categorized as Non-Hispanic White, Non-Hispanic Black, Hispanic or Mexican American, and Non-Hispanic Asian. Education was self-reported and categorized as “high school graduate or less” or “some college education or above”. Marital status was self-reported and categorized as “married or living with a partner”, “widowed, divorced, or separated”, and “never married”. Poverty-to-Income ratio, or the self-reported annual family income divided by the poverty threshold, was categorized into less than or equal to 1.29, 1.30 to 3.49, and 3.50 or above.

#### Body mass index

Height and weight were measured by trained personnel using standardized procedures and calibrated equipment. BMI (kg/m^2^) was calculated as weight in kilograms divided by the square of height in meters and was categorized as healthy weight (BMI 18.5 kg/m^2^ to < 25 kg/m^2^), overweight (BMI 25 kg/m^2^ to < 30 kg/m^2^), and obese (BMI ≥ 30 kg/m^2^).

#### Smoking status

Smoking status was assessed from two self-reported items in the data: “Have you smoked at least 100 cigarettes in your lifetime?” and “Do you smoke cigarettes now?” Smoking status was categorized into three groups: (1) never smokers who reported they have not smoked 100 cigarettes in their lifetime and do not smoke now; former smokers who reported they have smoked at least 100 cigarettes in their lifetime, but do not smoke now; and (3) current smokers who reported they smoked at least 100 cigarettes in their lifetime and currently smoke either every day or some days.

#### Alcohol use

Participants were asked if they had at least 12 drinks of alcohol in the past year and in their lifetime. Similar to Muntner et al., alcohol status was categorized into three categories: (1) never drinkers who reported no alcohol drinking or consumed less than 12 alcoholic drinks in their lifetime and in the past year; (2) former drinkers who reported drinking at least 12 alcoholic drinks in their lifetime but none in the last year; and (3) current drinkers who reported drinking at least 12 alcoholic drinks in their lifetime and had at least 12 alcoholic drinks in the last year [19].

#### Physical activity

Participants reported the number of days and amount of time spent participating in moderate or vigorous activity at work or as recreation. Physical activity was calculated into metabolic equivalent of task (MET), the ratio between caloric consumption during physical activity and resting basal metabolic rate. MET were calculated by using the MET value of the activity and multiplying by the duration of the activity in minutes. The validity of self-reported physical activity measure, indexed using MET-minutes, has been demonstrated previously [20]. Participants were categorized into four groups, according to the US Physical Activity Guidelines for Americans [21]. Participants who reported no physical activity were categorized as sedentary. Those who reported physical activity equal to less than 500 MET-minutes per week were categorized as “Low”. Participants who reported ≥ 500 and < 1000 MET-minutes per week were categorized as “Moderate”. Physical activity levels ≥ 1000 MET-minutes per week were categorized as “High” [21].

#### Diet

Dietary intake was assessed by two 24-hr dietary recalls. Based on Mellen et al., a dietary index or DASH score was generated based on the average target values for 9 nutrients: total fat, saturated fat, protein, fiber, cholesterol, calcium, magnesium, sodium, and potassium [22, 23]. Individuals who met the goal for each nutrient received 1 point, those who met an intermediate goal received half of a point, and those who did not meet any goals received 0 points. The total score ranges from 0 to 9 points. Individuals meeting approximately half of the DASH targets (DASH score ≥ 4.5) were considered DASH accordant [23].

### Statistical analysis

All analyses were performed using NHANES-generated sampling strata, clusters, and weights to generate nationally representative estimates in US civilian population. Categorical variables were presented as percentages, and continuous variables were presented as means with standard deviation. We examined group differences in sample characteristics by status using Chi-square or t-test. We used weighted logistic regression to estimate the presence of MetS and each of its components to CD and CS status, compared to NCD and stratified by gender. In addition, we repeated the logistic regression analysis to estimate the association between CS cancer site and presence of MetS and each component, compared to NCD. All multivariable logistic regression models were adjusted for demographic characteristics such as age, race/ethnicity, education, PIR, BMI, physical activity, DASH score, smoking status, and alcohol use [24, 25]. Adjusted estimates of association were expressed as odds ratios with 95% confidence intervals. A two-sided *P* value < 0.05 was considered statistically significant. Stata version 17.0 SE was used for all analyses.

## Results

### Characteristics of the study sample

Table 1 describes the characteristics of participants included in this study. Our study sample included 12,734 participants (52.8% women, 72.3% non-Hispanic White, and mean age 58.5 [10.2 SD] years), among whom 4,494 were NCD (36.5%), 6493 CD (47.5%), and 1747 CS (16.0%). In the total sample, most participants (72.5%) met the criteria for MetS. Across study groups, there were statistically significant differences in all demographic factors including gender, average age, race and ethnicity, marital status, education, and poverty-to-income ratio. Compared to CD and CS, most NCD were women, younger, married or living with a partner, and belonged to the highest PIR group (Table 1).

**Table 1:** Characteristics of study participants by NCD, CD, and CS status

	NCD (n = 4,494)	CD (n = 6,493)	CS (n = 1,747)	Total (n = 12,734)
Gender, % ^***^				
*Women*	51.1	53.8	54.0	52.8
*Men*	48.9	46.2	46.0	47.2
Mean age (sd) ^***^	53.1 (8.4)	60.1 (10.0)	65.7 (9.5)	58.5 (10.2)
Race/Ethnicity, % ^***^				
*Non-Hispanic White*	66.9	71.5	87.3	72.3
*Non-Hispanic Black*	11.5	12.0	5.6	10.8
*Hispanic*	14.4	12.4	5.1	12.0
*Non-Hispanic Asian*	7.2	4.1	2.0	4.9
Marital status, % ^***^				
*Married/living with partner*	72.0	64.6	66.4	67.6
*Widowed/divorced/separated*	19.6	27.7	29.9	25.1
*Never married*	8.4	7.8	3.7	7.3
Education, % ^***^				
*High school graduate or less*	34.1	41.3	28.1	36.5
*Some college or above*	65.9	58.7	71.9	63.5
Poverty-Income-Ratio, % ^***^				
*< 1.29*	13.9	22.6	13.5	18.0
*1.30–3.49*	32.3	35.8	36.5	34.6
*≥* *3.50*	53.8	41.6	50.0	47.4
BMI category (kg/m^2^), % ^***^				
*Healthy (18.5–24.9)*	29.9	18.9	25.9	24.0
*Overweight (25.0–29.9)*	38.8	30.8	34.4	34.3
*Obese (≥* *30.0)*	31.3	50.3	39.7	41.7
Total Physical Activity (METs), % ^***^				
*Sedentary*	18.7	29.5	28.7	25.4
*Low (≤* *499)*	13.1	14.4	14.1	13.9
*Moderate (500–999)*	11.5	9.8	11.3	10.6
*High (≥* *1000)*	56.8	46.3	45.9	50.1
Smoking, % ^***^				
*Never smoker*	62.4	48.6	46.6	53.3
*Former smoker*	23.5	32.2	39.4	30.1
*Current smoker*	14.1	19.3	14.1	16.6
Alcohol use, % ^***^				
*Never drinker*	10.5	10.8	8.6	10.4
*Former drinker*	10.7	16.2	16.2	14.2
*Current drinker*	78.8	73.0	75.0	75.4
DASH accordant, %	11.4	9.2	8.9	10.0

Abbreviations: NCD, no self-reported medical condition; CD, 1 or more self-reported medical conditions; CS, cancer survivor; BMI, body mass index; MET, metabolic equivalent of task.

DASH accordant: DASH score ≥ 4.5.

* *p* < 0.05, ** *p* < 0.01, *** *p* < 0.001

### Cancer survivor status and MetS

In multivariable adjusted logistic regression analysis (Table 2), compared to NCD men, CS men had 2.6-fold higher odds (OR 2.60; CI 1.75–3.87) and CD men had 2.2-fold higher odds (OR 2.18; CI 1.59–2.98) of meeting MetS. Both groups also demonstrated higher odds of meeting 4 to 5 MetS component compared to NCD men, particularly elevated WC (OR 3.81; CI 2.41–6.04) and low HDL-c (OR 2.52; CI 1.81–3.52) in CS men. CS and CD women also had higher odds of meeting MetS criteria compared to their healthy counterparts, OR 2.05 (CI 1.44–2.93) and OR 2.14 (CI 1.63–2.81) respectively.

**Table 2:** Odds ratiosa (95% CI) for MetS and its components among CD and CS participants, stratified by sex/gender

	MetS	Elevated WC	Low HDL-c	Elevated TG	Elevated FG	Elevated BP
Men						
NCD	Ref	Ref	Ref	Ref	Ref	Ref
CD	2.18^***^ [1.59,2.98]	2.11^***^ [1.48,3.02]	2.48^***^ [1.85,3.31]	1.78^**^ [1.27,2.51]	1.55^*^ [1.07,2.23]	1.90^***^ [1.43,2.52]
CS	2.60^***^ [1.75,3.87]	3.81^***^ [2.41,6.04]	2.52^***^ [1.81,3.52]	2.40^***^ [1.61,3.60]	1.60 [0.96,2.69]	1.95^***^ [1.49,2.56]
Women						
NCD	Ref	Ref	Ref	Ref	Ref	Ref
CD	2.14^***^ [1.63,2.81]	1.80^***^ [1.33,2.44]	1.97^***^ [1.57,2.46]	1.34^*^ [1.04,1.71]	1.08 [0.81,1.45]	1.79^***^ [1.38,2.33]
CS	2.05^***^ [1.44,2.93]	1.93^***^ [1.35,2.76]	1.79^***^ [1.31,2.44]	1.47^*^ [1.05,2.04]	1.65^*^ [1.08,2.50]	1.68^**^ [1.23,2.28]

Abbreviations: MetS, Metabolic syndrome; CS, cancer survivor; CD, 1 or more self-reported medical conditions; WC, waist circumference; HDL-c, high-density lipoprotein cholesterol; TG, triglycerides; FG, fasting plasma glucose; BP, blood pressure.

Elevated WC, ≥ 102 cm for men, ≥ 88 cm for women

Low HDL-c, < 40 mg/dL for men and < 50 mg/dL for women, or current treatment for reduced HDL-c

Elevated TG, ≥ 150 mg/dL or current treatment for elevated triglycerides.

Elevated FG (≥ 100 mg/dL) or current use of diabetes medication.

Elevated BP, systolic blood pressure ≥ 130, or diastolic blood pressure ≥ 85, or current use of blood pressure medication.

^a^Adjusted by age, race/ethnicity, education, PIR, BMI, physical activity, DASH score, smoking status, and alcohol use.

^*^p < 0.05, ^**^p < 0.01, ^***^p < 0.001

### Cancer site and MetS

Among CS men (Table 3), those with a history of blood-related cancer (OR 4.88, CI 1.30–18.37), colorectal cancer (OR 2.87, CI 1.02–8.11), prostate cancer (OR 2.85; CI 1.51–5.38), or non-melanoma skin cancer (OR 2.58; CI 1.37–4.88) were associated with MetS. In analysis of MetS components, prostate cancer, melanoma, non-melanoma skin cancer, and bladder cancer demonstrated statistically significant association with at least three of the five MetS components, elevated WC, low HDL-c, and elevated TG. In CS women (Table 4), those with cervical (OR 4.25; CI 1.70–10.59), melanoma (OR 3.51; CI 1.28–9.59), and breast cancer (OR 1.76; CI 1.08–2.89) showed statistically significant association with MetS compared to NCD women. In contrast to CS men, few cancer sites among CS women exhibited a statistically significant association with individual MetS components, compared to their healthy counterparts.

**Table 3:** Odds ratiosa (95% CI) for MetS and its components in CS and CD men by cancer site compared to healthy men

	MetS	Elevated WC	Low HDL-c	Elevated TG	Elevated FG	Elevated BP
NCD	Ref	Ref	Ref	Ref	Ref	Ref
CD	2.18^***^ [1.59,2.98]	2.13^***^ [1.48,3.05]	2.48^***^ [1.86,3.31]	1.79^**^ [1.27,2.52]	1.56^*^ [1.09,2.24]	1.90^***^ [1.42,2.52]
Prostate	2.85^**^ [1.51,5.38]	3.58^***^ [1.87,6.85]	2.14^**^ [1.22,3.77]	1.97^*^ [1.02,3.79]	1.60 [0.82,3.14]	2.44^**^ [1.42,4.18]
Colorectal	2.87^*^ [1.02,8.11]	12.30^***^ [4.12,36.70]	4.83^***^ [1.99,11.74]	1.25 [0.37,4.24]	4.91 [0.64,37.38]	1.86 [0.61,5.64]
Melanoma	2.71 [0.73,10.09]	4.52^**^ [1.54,13.26]	5.42^**^ [1.97,14.93]	3.77^*^ [1.24,11.40]	3.56 [0.95,13.43]	1.98 [0.75,5.23]
Non-melanoma	2.58^**^ [1.37,4.88]	5.17^***^ [2.44,10.95]	1.96^*^ [1.16,3.31]	2.52^**^ [1.27,5.01]	1.49 [0.69,3.21]	1.72 [0.98,3.02]
Blood-related	4.88^*^ [1.30,18.37]	1.67 [0.41,6.85]	3.60 [0.94,13.79]	1.73 [0.50,5.95]	1.44 [0.30,6.83]	2.05 [0.45,9.29]
Bladder	3.17 [0.95,10.62]	6.40^**^ [1.73,23.69]	2.78^*^ [1.07,7.18]	4.45^*^ [1.39,14.25]	2.08 [0.40,10.79]	0.76 [0.27,2.15]
Other GU	1.51 [0.43,5.21]	2.63 [0.70,9.83]	2.42 [0.81,7.26]	8.45^*^ [1.51,47.26]	0.94 [0.17,5.22]	1.10 [0.45,2.67]
Other	2.31 [0.99,5.39]	3.38^**^ [1.43,7.98]	2.73^**^ [1.33,5.60]	1.92 [0.85,4.31]	2.97 [0.96,9.15]	1.45 [0.61,3.46]

Abbreviations: MetS, Metabolic syndrome; CS, cancer survivor; GU, genitourinary cancer; WC, waist circumference; HDL-c, high-density lipoprotein cholesterol; TG, triglycerides; FG, fasting plasma glucose; BP, blood pressure.

Elevated WC, ≥ 102 cm for men, ≥ 88 cm for women

Low HDL-c, < 40 mg/dL for men and < 50 mg/dL for women, or current treatment for reduced HDL-c

Elevated TG, ≥ 150 mg/dL or current treatment for elevated triglycerides.

Elevated FG (≥ 100 mg/dL) or current use of diabetes medication.

Elevated BP, systolic blood pressure ≥ 130, or diastolic blood pressure ≥ 85, or current use of blood pressure medication.

^a^Adjusted by age, race/ethnicity, education, PIR, BMI, physical activity, DASH score, smoking status, and alcohol use.

^*^p < 0.05, ^**^p < 0.01, ^***^p < 0.001

**Table 4:** Odds ratiosa (95% CI) for MetS and its components in CS and CD women by cancer site compared to healthy women

	MetS	Elevated WC	Low HDL-c	Elevated TG	Elevated FG	Elevated BP
NCD	Ref	Ref	Ref	Ref	Ref	Ref
CD	2.15^***^ [1.64,2.82]	1.81^***^ [1.34,2.44]	1.98^***^ [1.58,2.48]	1.34^*^ [1.05,1.72]	1.08 [0.80,1.45]	1.79^***^ [1.38,2.33]
Breast	1.76^*^ [1.08,2.89]	2.22^*^ [1.15,4.30]	1.49 [0.96,2.31]	1.33 [0.78,2.25]	1.51 [0.95,2.40]	1.59^*^ [1.03,2.43]
Colorectal	1.89 [0.79,4.51]	2.30 [0.47,11.22]	1.33 [0.74,2.42]	1.54 [0.60,3.99]	1.11 [0.46,2.72]	2.37 [0.49,11.46]
Melanoma	3.51^*^ [1.28,9.59]	3.48^*^ [1.33,9.08]	1.32 [0.62,2.84]	1.07 [0.36,3.13]	7.94^**^ [2.02,31.23]	1.49 [0.59,3.77]
Non-melanoma	1.68 [0.81,3.49]	1.87 [0.79,4.40]	1.88 [0.97,3.66]	1.62 [0.82,3.21]	1.24 [0.54,2.83]	2.32^*^ [1.20,4.48]
Blood-related	2.30 [0.49,10.74]	4.22^*^ [1.05,16.92]	1.84 [0.57,5.97]	2.19 [0.48,9.93]	3.54 [0.42,30.03]	1.10 [0.27,4.51]
Cervical	4.25^**^ [1.70,10.59]	1.26 [0.54,2.91]	2.05 [0.98,4.27]	1.30 [0.48,3.53]	1.74 [0.68,4.48]	1.50 [0.65,3.43]
Uterine	2.58 [0.66,10.00]	2.76 [0.91,8.39]	2.05 [0.70,5.98]	2.66 [0.52,13.61]	2.10 [0.77,5.73]	1.15 [0.34,3.85]
Other	1.78 [0.89,3.53]	1.86 [0.87,3.94]	2.23^**^ [1.26,3.95]	1.17 [0.59,2.34]	1.38 [0.58,3.26]	1.28 [0.73,2.24]

Abbreviations: MetS, Metabolic syndrome; CS, cancer survivor; WC, waist circumference; HDL-c, high-density lipoprotein cholesterol; TG, triglycerides; FG, fasting plasma glucose; BP, blood pressure.

Elevated WC, ≥ 102 cm for men, ≥ 88 cm for women

Low HDL-c, < 40 mg/dL for men and < 50 mg/dL for women, or current treatment for reduced HDL-c

Elevated TG, ≥ 150 mg/dL or current treatment for elevated triglycerides.

Elevated FG (≥ 100 mg/dL) or current use of diabetes medication.

Elevated BP, systolic blood pressure ≥ 130, or diastolic blood pressure ≥ 85, or current use of blood pressure medication.

^a^Adjusted by age, race/ethnicity, education, PIR, BMI, physical activity, DASH score, smoking status, and alcohol use.

^*^p < 0.05, ^**^p < 0.01, ^***^p < 0.001

## Discussion

### Summary of main findings

Compared to their NCD counterparts, CS men and women demonstrated higher odds of meeting MetS criteria and at least three of the five individual MetS components than CD. In particular, men showed a stronger association with MetS than women. Furthermore, certain cancer sites in CS men and women had statistically significant higher odds of MetS and its components, compared to their healthy counterparts. In CS men, participants with a history of hematologic malignancies had the highest odds of developing MetS compared to NCD men. For CS women, participants with cervical cancer history exhibited the highest odds of MetS than their healthy counterparts. In subgroup analyses of the individual components of MetS, CS men with cancer sites associated with MetS had higher odds of having elevated WC, low HDL-c, and elevated TG compared to NCD men.

### Comparison with prior studies and pathogenesis of MetS among cancer survivors

In CS men, the pathogenesis of MetS can be related to the changes in endocrine and metabolic functions due to treatment. Similar to this study, CS men with a history of hematologic malignancies have been shown to have a high risk of developing MetS, which has been attributed to extensive treatments such as stem cell transplantation, high dose chemotherapy, and irradiation [26, 27]. These treatments cause damage to the hypothalamic-pituitary-axis, leading to deficiencies in growth hormones, thyroid hormones, and androgens related to individual components of MetS [28]. For prostate cancer survivors, androgen deprivation therapy (ADT), a common treatment modality, has also been proposed as a risk factor for MetS development, due to male hypogonadism or low testosterone. In one study, patients who received ADT had higher prevalence of MetS (55%) than patients treated with radiotherapy and/or prostatectomy (22%), or healthy controls (20%) [5, 29]. Furthermore, observational studies have shown a positive association between level of hypogonadism and degree of obesity in men, as well as an inverse relationship between testosterone levels and visceral fat mass [30]. Low testosterone has also been linked to adverse lipid profile [31] and hypertension [32, 33], two components of MetS.

For CS women, previous epidemiological studies have also supported correlation between MetS and a history of cervical cancer [34, 35]. Penaranda et al., showed that CS women with cervical cancer history had higher odds of MetS in both unadjusted and adjusted analyses (which accounted for multiple sexual partners, multiparity, hormonal contraceptive use, and history of smoking) compared to those without MetS [35]. A limited number studies have investigated the relationship between a history of cervical cancer and MetS, but it is hypothesized that MetS can be related to low estrogen (e.g. caused by direct damage to the ovaries from abdomino-pelvic radiotherapy), abdominal adiposity, and increased inflammatory markers, such as adipokines and other cytokines, from persistent human papillomavirus infection [5, 36]. Moreover, in our study, none of the specific MetS components were significantly associated with cervical cancer in CS women. Moreover, there were very few statistically significant associations between individual MetS components and other cancer sites in CS women. Synergism, or clustering of MetS components, may be necessary for the development of MetS, and long-term risk may be underestimated at any point in time in CS women.

Individual components of MetS have been reported to be carcinogenic, which may lead to increased risk in recurrence in CS. In subgroup analysis of MetS components, most CS men had higher odds of having elevated WC, low HDL-c, and elevated TG compared to NCD men. Studies have repeatedly reported that components of MetS related to hyperglycemia, obesity, and dyslipidemia promote cancer development as well as growth [2, 5]. Epidemiological studies have revealed that an elevated WC, BMI, or waist-hip-ratio, indicators of obesity, are linked with several cancer types and cancer-related mortality [10, 37, 38]. Elevated WC has been shown to be a major determinant of higher prevalence of MetS in prostate cancer survivors [5, 29]. Conversely, MetS has been theorized to be a major risk factor for prostate cancer development. In a prospective cohort of 16,209 men aged 40–49 years, men in the upper quartile for two components of MetS were 23% more likely to be diagnosed with prostate cancer, and those who met three criteria for MetS were 56% more likely to be diagnosed with prostate cancer compared to the rest of the cohort [39]. In the pathophysiology of obesity and cancer, hyperinsulinemia related to insulin resistance increases the bioavailability of insulin-like growth factor (IGF)-1 [10, 40]. Receptors for insulin and IGF-1 are expressed in most cancer cells, and receptor activation results in signaling pathways capable of stimulating cancer cell proliferation, protection from apoptotic stimuli, invasion and metastasis [40]. Low HDL-c and elevated TG have also been demonstrated to be risk factors for multiple cancers, including colon cancer, melanoma, and prostate cancer [41]. For example, low HDL-c was reported to be related to an increase in lung cancer incidence and associated with a 15-fold increase in hematologic malignancy development [42]. In addition, hypertriglyceridemia has been reported to be associated with prostate cancer [43]. The probable mechanism through which elevated triglycerides increase cancer risk is through the generation of reactive oxygen species and oxidative stress, which cause DNA damage [10]. Mechanisms that may link low HDL-c with cancer are not well understood. One possible mechanism may involve chronic inflammation, resulting from decreased HDL-c levels, which has been implicated in cancer development [10, 44].

MetS is a common long-term complication of cancer that affects health outcomes and quality of life. The development of the MetS in cancer survivors has been associated with signs of early atherosclerosis and cancer recurrence. Considering the direct cardiovascular toxicity of most cancer treatments, this group faces an increased risk of cardiovascular disease, and the presence of MetS provides the environment for development of secondary cancers. Our study also highlights the gender differences in risk of MetS and, consequently, cardiovascular disease, which may be of potential relevance for prevention. With prolonged cancer survival rates, prevalence of MetS is expected to increase, and therefore, it is important for healthcare professionals to acknowledge the risk of MetS and establish suitable screening, follow-up and appropriate interventions.

### Strengths and limitations

The strengths of this study include a large sample size providing enough statistical power to conduct stratified analyses, and the use of a representative sample of the US population, providing results can be extrapolated to the population using US census data. Our multivariable analysis included a robust adjustment using wide variety of covariates including dietary intake and physical activity. All anthropometric or biochemical components of MetS were objectively measured with validated tools.

This study also has a few limitations. As a cross-sectional study, it is difficult to draw causal inference between cancer status and MetS or each of its components, as well as to establish the temporal sequence between cancer status and MetS. These results should be considered hypothesis-generating, which requires prospective studies to further characterized the risk of MetS among cancer survivors. Other limitations in this study are inherent to the use of NHANES dataset, which includes nonresponse bias, recall bias, and inability to differentiate between sex and gender. Although NHANES collects data on a wide variety of variables, there are unmeasured confounders such as type of cancer treatment, a factor implicated in development of MetS, and years since treatment completion. It is also difficult to draw definitive conclusions given that several definitions of MetS exist, and we used only one of these. A gold standard for MetS diagnosis has not been established and each definition emphasizes different aspects of MetS, which may explain the differences in results in various studies.

## Conclusion

With an aging US population and concurrent increases in chronic conditions, prevalence of MetS is increasing and becoming a major concern. Detecting and controlling the components of MetS, especially the preeminent ones, may improve length and quality of life for cancer survivors. Furthermore, efforts aimed at improving lifestyle patterns including physical activity and dietary habits or use of medications, which can reduce metabolic aberrations, can have profound health benefits for cancer survivors.

## References

[1] Meigs JB, et al. Impact of insulin resistance on risk of type 2 diabetes and Cardiovascular Disease in People with metabolic syndrome. Diabetes Care. 2007;30(5):1219–25. 17259468 10.2337/dc06-2484

[2] Esposito K, et al. Metabolic syndrome and risk of cancer: a systematic review and meta-analysis. Diabetes Care. 2012;35(11):2402–11. 23093685 10.2337/dc12-0336 PMC3476894

[3] Zimmet PZ, Alberti KGM. Introduction: globalization and the non-communicable disease epidemic. Obesity. 2006;14(1):1. 16493116 10.1038/oby.2006.1

[4] Ogden CL, et al. Prevalence of overweight and obesity in the United States, 1999–2004. JAMA. 2006;295(13):1549–55. 16595758 10.1001/jama.295.13.1549

[5] de Haas EC, et al. The metabolic syndrome in cancer survivors. Lancet Oncol. 2010;11(2):193–203. 20152771 10.1016/S1470-2045(09)70287-6

[6] Aguilar M, et al. Prevalence of the metabolic syndrome in the United States, 2003–2012. JAMA. 2015;313(19):1973–4. 25988468 10.1001/jama.2015.4260

[7] Hirode G, Wong RJ. Trends in the prevalence of metabolic syndrome in the United States, 2011–2016. JAMA. 2020;323(24):2526–8. 32573660 10.1001/jama.2020.4501 PMC7312413

[8] Pucci G, et al. Sex- and gender-related prevalence, cardiovascular risk and therapeutic approach in metabolic syndrome: a review of the literature. Pharmacol Res. 2017;120:34–42. 28300617 10.1016/j.phrs.2017.03.008

[9] Gathirua-Mwangi WG, et al. Metabolic syndrome and total cancer mortality in the Third National Health and Nutrition Examination Survey. Cancer Causes Control. 2017;28(2):127–36. 28097473 10.1007/s10552-016-0843-1 PMC5308139

[10] Cowey S, Hardy RW. The metabolic syndrome: a high-risk state for cancer? Am J Pathol. 2006;169(5):1505–22. 17071576 10.2353/ajpath.2006.051090 PMC1780220

[11] Miller KD et al. *Cancer treatment and survivorship statistics, 2019* CA: A Cancer Journal for Clinicians, 2019. 69(5): p. 363–385. 10.3322/caac.21565 31184787

[12] Statistics NCfH. *National Health and Nutrition Examination Survey*; Available from: http://www.cdc.gov/nchs/nhanes.htm.

[13] Pluimakers VG, et al. Metabolic syndrome as cardiovascular risk factor in childhood cancer survivors. Crit Rev Oncol Hematol. 2019;133:129–41. 30661649 10.1016/j.critrevonc.2018.10.010

[14] Chueh HW, Yoo JH. Metabolic syndrome induced by anticancer treatment in childhood cancer survivors. Ann Pediatr Endocrinol Metab. 2017;22(2):82–9. 28690985 10.6065/apem.2017.22.2.82 PMC5495983

[15] Scott JX, Latha MS, Aruna R. Approach to metabolic syndrome in childhood cancer survivors. Indian J Cancer. 2015;52(2):169–72. 26853389 10.4103/0019-509X.175840

[16] Zhao H, et al. Niacin, lutein and zeaxanthin and physical activity have an impact on Charlson comorbidity index using zero-inflated negative binomial regression model: National Health and Nutrition Examination Survey 2013–2014. BMC Public Health. 2019;19(1):1589. 31779602 10.1186/s12889-019-7906-7 PMC6883694

[17] Charlson ME, et al. A new method of classifying prognostic comorbidity in longitudinal studies: development and validation. J Chronic Dis. 1987;40(5):373–83. 3558716 10.1016/0021-9681(87)90171-8

[18] Grundy SM, et al. Diagnosis and management of the metabolic syndrome: an American Heart Association/National Heart, Lung, and Blood Institute Scientific Statement. Circulation. 2005;112(17):2735–52. 16157765 10.1161/CIRCULATIONAHA.105.169404

[19] Muntner P, et al. Prevalence of non-traditional cardiovascular disease risk factors among persons with impaired fasting glucose, impaired glucose tolerance, diabetes, and the metabolic syndrome: analysis of the Third National Health and Nutrition Examination Survey (NHANES III). Ann Epidemiol. 2004;14(9):686–95. 15380800 10.1016/j.annepidem.2004.01.002

[20] Kyu HH et al. *Physical activity and risk of breast cancer, colon cancer, diabetes, ischemic heart disease, and ischemic stroke events: systematic review and dose-response meta-analysis for the Global Burden of Disease Study* 2013. bmj, 2016. 354. 10.1136/bmj.i3857 PMC4979358 27510511

[21] Services U. S.D.o.H.a.H., *Physical Activity Guidelines for Americans, 2nd Edition*. 2018, U.S. Department of Health and Human Services: Washington, DC.

[22] Mellen PB, et al. Deteriorating dietary habits among adults with hypertension: DASH dietary accordance, NHANES 1988–1994 and 1999–2004. Arch Intern Med. 2008;168(3):308–14. 18268173 10.1001/archinternmed.2007.119

[23] Miller PE, et al. Comparison of 4 established DASH diet indexes: examining associations of index scores and colorectal cancer. Am J Clin Nutr. 2013;98(3):794–803. 23864539 10.3945/ajcn.113.063602 PMC3743737

[24] Park Y-W, et al. The metabolic syndrome: prevalence and Associated Risk factor findings in the US Population from the Third National Health and Nutrition Examination Survey, 1988–1994. Arch Intern Med. 2003;163(4):427–36. 12588201 10.1001/archinte.163.4.427 PMC3146257

[25] Dallongeville J, et al. Household Income is Associated with the risk of metabolic syndrome in a sex-specific manner. Diabetes Care. 2005;28(2):409–15. 15677801 10.2337/diacare.28.2.409

[26] Annaloro C, et al. Prevalence of metabolic syndrome in long-term survivors of hematopoietic stem cell transplantation. Bone Marrow Transplant. 2008;41(9):797–804. 18195686 10.1038/sj.bmt.1705972

[27] Majhail NS, et al. High prevalence of metabolic syndrome after allogeneic hematopoietic cell transplantation. Bone Marrow Transplant. 2009;43(1):49–54. 18724397 10.1038/bmt.2008.263 PMC2628412

[28] Li C, et al. Metabolic syndrome in hematologic malignancies survivors: a meta-analysis. Med Oncol. 2014;32(1):422. 25471791 10.1007/s12032-014-0422-9

[29] Braga-Basaria M, et al. Metabolic syndrome in men with prostate Cancer undergoing long-term androgen-deprivation therapy. J Clin Oncol. 2006;24(24):3979–83. 16921050 10.1200/JCO.2006.05.9741

[30] Dandona P, Dhindsa S. Update: hypogonadotropic hypogonadism in type 2 diabetes and obesity. J Clin Endocrinol Metab. 2011;96(9):2643–51. 21896895 10.1210/jc.2010-2724 PMC3167667

[31] Haffner SM, et al. Relationship of sex hormones to lipids and lipoproteins in nondiabetic men. J Clin Endocrinol Metabolism. 1993;77(6):1610–5. 10.1210/jcem.77.6.8263149 8263149

[32] Khaw K-T, Barrett-Connor E. Blood pressure and endogenous testosterone in men: an inverse relationship. J Hypertens. 1988;6(4):329–32. 3379300

[33] Dockery F, et al. Testosterone suppression in men with prostate cancer leads to an increase in arterial stiffness and hyperinsulinaemia. Clin Sci. 2003;104(2):195–201. 10.1042/CS20020209 12546642

[34] Ulmer H, et al. Metabolic risk factors and cervical cancer in the metabolic syndrome and cancer project (Me–Can). Gynecol Oncol. 2012;125(2):330–5. 22330614 10.1016/j.ygyno.2012.01.052

[35] Penaranda EK, Shokar N, Ortiz M. *Relationship between metabolic syndrome and history of cervical cancer among a US national population* International Scholarly Research Notices, 2013. 2013.

[36] Baker R, et al. Increased plasma levels of adipokines and inflammatory markers in older women with persistent HPV infection. Cytokine. 2011;53(3):282–5. 21167737 10.1016/j.cyto.2010.11.014 PMC3033991

[37] Calle EE, Kaaks R. Overweight, obesity and cancer: epidemiological evidence and proposed mechanisms. Nat Rev Cancer. 2004;4(8):579–91. 15286738 10.1038/nrc1408

[38] Renehan AG, et al. Body-mass index and incidence of cancer: a systematic review and meta-analysis of prospective observational studies. The lancet. 2008;371(9612):569–78. 10.1016/S0140-6736(08)60269-X 18280327

[39] Lund Håheim L, et al. Metabolic syndrome predicts prostate Cancer in a cohort of Middle-aged norwegian men followed for 27 years. Am J Epidemiol. 2006;164(8):769–74. 16952929 10.1093/aje/kwj284

[40] Uzunlulu M, Telci Caklili O, Oguz A. Association between Metabolic Syndrome and Cancer. Annals of Nutrition and Metabolism. 2016;68(3):173–9. 26895247 10.1159/000443743

[41] Borena W, et al. Serum triglycerides and cancer risk in the metabolic syndrome and cancer (Me-Can) collaborative study. Cancer Causes Control. 2011;22(2):291–9. 21140204 10.1007/s10552-010-9697-0

[42] Shor R, et al. Low serum LDL cholesterol levels and the risk of fever, sepsis, and malignancy. Ann Clin Lab Sci. 2007;37(4):343–8. 18000291

[43] Wuermli L, et al. Hypertriglyceridemia as a possible risk factor for prostate cancer. Prostate Cancer Prostatic Dis. 2005;8(4):316–20. 16158078 10.1038/sj.pcan.4500834

[44] Ganjali S, et al. HDL and cancer - causality still needs to be confirmed? Update 2020. Sem Cancer Biol. 2021;73:169–77. 10.1016/j.semcancer.2020.10.007 33130036

